# Early Activation of MAPK p44/42 Is Partially Involved in DON-Induced Disruption of the Intestinal Barrier Function and Tight Junction Network

**DOI:** 10.3390/toxins8090264

**Published:** 2016-09-08

**Authors:** Alexandra Springler, Sabine Hessenberger, Gerd Schatzmayr, Elisabeth Mayer

**Affiliations:** Biomin Research Center, Technopark 1, 3430 Tulln an der Donau, Austria; alexandra.springler@biomin.net (A.S.); sabine.hessenberger@biomin.net (S.H.); gerd.schatzmayr@biomin.net (G.S.)

**Keywords:** transepithelial electrical resistance, mycotoxin, IPEC-J2, tight junctions, deoxynivalenol, de-epoxy deoxynivalenol

## Abstract

Deoxynivalenol (DON), produced by the plant pathogens *Fusarium* *graminearum* and *Fusarium culmorum*, is one of the most common mycotoxins, contaminating cereal and cereal-derived products. Although worldwide contamination of food and feed poses health threats to humans and animals, pigs are particularly susceptible to this mycotoxin. DON derivatives, such as deepoxy-deoxynivalenol (DOM-1), are produced by bacterial transformation of certain intestinal bacteria, which are naturally occurring or applied as feed additives. Intestinal epithelial cells are the initial barrier against these food- and feed-borne toxins. The present study confirms DON-induced activation of MAPK p44/42 and inhibition of p44/42 by MAPK-inhibitor U0126 monoethanolate. Influence of DON and DOM-1 on transepithelial electrical resistance (TEER), viability and expression of seven tight junction proteins (TJ), as well as the potential of U0126 to counteract DON-induced effects, was assessed. While DOM-1 showed no effect, DON significantly reduced TEER of differentiated IPEC-J2 and decreased expression of claudin-1 and -3, while leaving claudin-4; ZO-1, -2, and -3 and occludin unaffected. Inhibition of p44/42 counteracted DON-induced TEER decrease and restored claudin-3, but not claudin-1 expression. Therefore, effects of DON on TEER and claudin-3 are at least partially p44/42 mediated, while effects on viability and claudin-1 are likely mediated via alternative pathways.

## 1. Introduction

The epithelial barrier of the gastrointestinal tract is the initial line of defense against orally ingested bacterial and viral pathogens, as well as food- and feed-derived toxins [1,2,3]. Structural integrity of the intestinal barrier, which is largely upheld by tight junctions, is essential for maintaining physiological gut functions. Tight junctions build a distinct network by sealing adjacent epithelial cells near the luminal surface. Not only does this regulate cell polarity, small molecule transport, and intestinal epithelial cell permeability, it also protects mucosal cells against harmful pathogens and toxic macromolecules [4].

An example of such a macromolecule is the *Fusarium*-derived trichothecene mycotoxin deoxynivalenol (DON), one of the most prevalent food- and feed-associated contaminants [5]. Due to its particularly high resistance to processing and heating [6,7], DON can readily enter the food and feed chain. Thus, the oral uptake of contaminated food or feed exposes intestinal epithelial cells to particularly high concentrations of this mycotoxin, causing impairments of the intestinal barrier function [8,9,10]. Upon cellular exposure, DON binds to the 60S ribosomal RNA peptidyltransferase site, leading to translational arrest and disruption of protein synthesis. Ribotoxic stress is induced, resulting in the activation of the mitogen-activated protein kinases (MAPK), which can be categorized into three subfamilies—namely p44/42 extracellular signal regulated kinase (ERK), p38 and c-Jun *N*-terminal Kinase (JNK). These regulate cell survival in response to stress, as well as physiological processes, including cell growth, differentiation and apoptosis [11]. Furthermore, MAPKs are crucial for signal transduction and immune response. With regard to the negative effect of DON on the intestinal barrier function, the MAPK p44/42 is of particular importance. It is involved in the regulation of intestinal epithelial cell morphology and tight junction structure, both of which regulate barrier function [9,12,13]. Numerous studies have demonstrated that MAPK p44/42 signaling can regulate paracellular transport across the intestinal lining, by up- or downregulating the expression of several tight junction proteins [14].

Studies have revealed a close relationship between DON exposure and the deterioration of the intestinal barrier, leading to increased paracellular permeability and the undesirable passage of xenobiotics, harmful molecules and pathogenic microorganisms across the intestinal lining. Particularly high DON susceptibility has been observed in pigs, whose diet is largely comprised of cereal-based feed [15].

Occurrence of DON is not only accompanied by other mycotoxins, but also by its derivatives, including those produced by fungi (3- and 15-acetyl-DON), plants (3-*O*-glucoside-DON), animals (DON-3 and DON-15-glucuronide and DON-sulfonates) or bacteria (3-keto-DON, 3-epi-DON, deepoxy-deoxynivalenol (DOM-1)) [16,17,18,19,20,21,22]. Polygastric animals and birds have high bacterial content located towards the top of the gastrointestinal tract, enabling DON to DOM-1 conversion prior to reaching the small intestine, where absorption of the mycotoxin takes place [23]. Although in monogastric animals the conversion of DON to DOM-1 is unlikely to occur prior to reaching the small intestine, relevance of DOM-1 nevertheless arises due to the use of a feed additive, containing DON to DOM-1 transforming bacteria (e.g., Gen. *nov*. (formerly Eubacterium) sp. *nov*. BBSH 797) [24]. Therefore, due to the natural and feed additive dependent exposure of intestinal cells to DOM-1, studies are required to elucidate relevance and safety of the metabolite with respect to intestinal barrier integrity. Thus far, this has been poorly documented. In fact, the amount of DON metabolites has not been considered in the regulatory limits fixed by food agencies, due to the lack of data regarding their absorption and toxicity [3,23]. The present study includes—in addition to the analysis of DON—a thorough investigation of the DON metabolite, DOM-1, with respect to barrier function of porcine intestinal epithelial cells, which has not yet been performed in this manner.

Therefore, the aim of this investigation was to provide a comprehensive overview of the effect of DON and its metabolite DOM-1 on barrier function of differentiated intestinal porcine epithelial cells (IPEC-J2). In contrast to cancer cell lines, IPEC-J2 exhibit strong morphological and functional resemblance to intestinal epithelial cells in vivo [25]. The study compares the effects of DON and DOM-1 on transepithelial electrical resistance and for the first time, provides an analysis of both mycotoxins on the expression of seven tight junction proteins. Furthermore, we determined the degree to which the detrimental effects of DON on TEER (transepithelial electrical resistance), viability and expression of tight junction proteins are due to the activation of MAPK p44/p42. The study extends current knowledge regarding toxicological effects of DON and its metabolite DOM-1, with particular focus on their effect on the barrier function of differentiated intestinal epithelial cells. Thus, the investigation provides important information regarding the safety of DON to DOM-1 transforming feed additives.

## 2. Results

### 2.1. Formation of a Differentiated IPEC-J2 Monolayer

**Figure 1:**
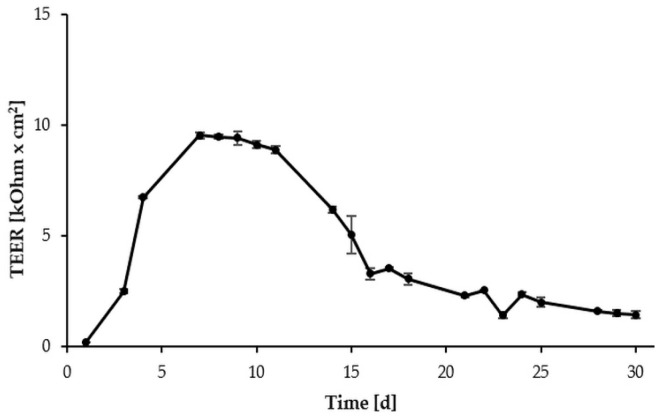
Progression of TEER (transepithelial electrical resistance) of intestinal porcine epithelial cells (IPEC-J2) seeded in 1.12 cm^2^ Transwell^®^ polyester membrane inserts, measured at indicated time points over a period of 30 days. Cells reached a steady-state TEER phase between Days 7 and 11. Data represent mean ± standard deviation (SD), *n* = 4.

To monitor monolayer formation, the progression of TEER of IPEC-J2, cultured in 1.12 cm^2^ Transwell^®^ polyester membrane inserts, was recorded over a period of 30 days. IPEC-J2 reached a resistance maximum after seven days of cultivation. At this time point, a well-established IPEC-J2 monolayer was observed microscopically. Between Day 7 (9.52 ± 0.15 kOhm × cm^2^) and Day 11 (8.88 ± 0.18 kOhm × cm^2^), TEER reached a steady-state phase (Figure 1), indicating a state of differentiation, suitable for toxin treatment.

### 2.2. Inhibition of DON-Induced p44/42 (ERK1/2) Activation by MAPK Inhibitor U0126 Monoethanolate (U0126)

**Figure 2:**
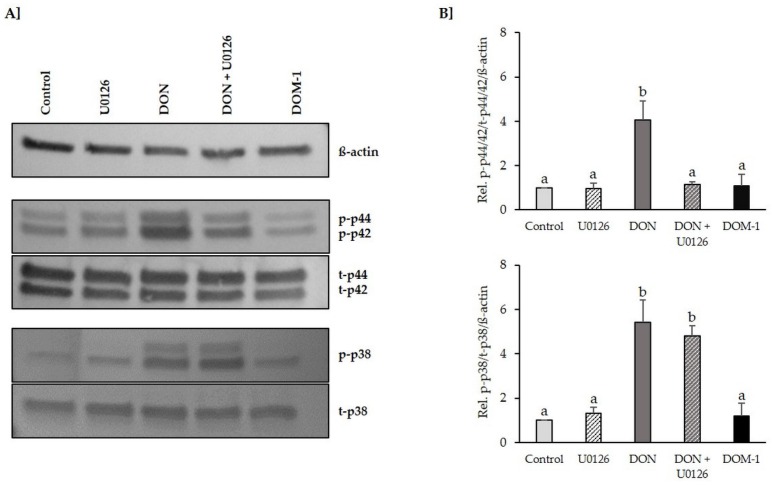
U0126 monoethanolate (U0126) (10 µM) counteracted deoxynivalenol (DON)-induced p44/42 mitogen activated protein kinase (MAPK) (Erk1/2), but not p38 MAPK activation. Differentiated IPEC-J2 were either pretreated with U0126 (10 µM) or complete cultivation medium before addition of DON (20 µM) for 1 h. Alternatively, differentiated IPEC-J2 were treated with deepoxy-deoxynivalenol (DOM-1) (100 µM) for 1 h. Phosphorylation of p44/42 MAPK (ERK1/2) (p-p44/42) and p38 MAPK (p-p38) was determined by (**A**) immunoblotting and (**B**) densitometry after normalization with endogenous signals (total p44/42 (t-p44/42) or total p38 (t-p38)) and ß-actin. Data were normalized to control and represent mean ± SD, *n* = 4. Statistically significant differences (*p* < 0.05) are indicated by different letters (a,b).

The ability of DON (20 µM) to trigger early activation of p44/42 MAPK (ERK1/2) and p38 MAPK signaling in differentiated IPEC-J2 was tested via immunoblotting (Figure 2). Treatment of IPEC-J2 with 20 µM DON for 1 h led to a significant activation of p44/42 MAPK (2.567 ± 0.33, *p* = 0.025) and p38 MAPK (4.11 ± 0.56, *p* = 0.000). DOM-1 (100 µM) did not affect the phosphorylation status of either MAPK protein. Pretreatment of differentiated IPEC-J2 with MAPK inhibitor U0126 (10 µM), followed by a 1 h DON exposure in the presence of U0126 (10 µM), prevented DON-induced p44/42 MAPK (ERK1/2) phosphorylation. DON-induced phosphorylation of p38 MAPK (3.15 ± 0.31, *p* = 0.000) was not affected by U0126 and remained significantly elevated compared to the control.

### 2.3. Effects of DON (+/− U0126) and DOM-1 on Intestinal Barrier Integrity

**Figure 3:**
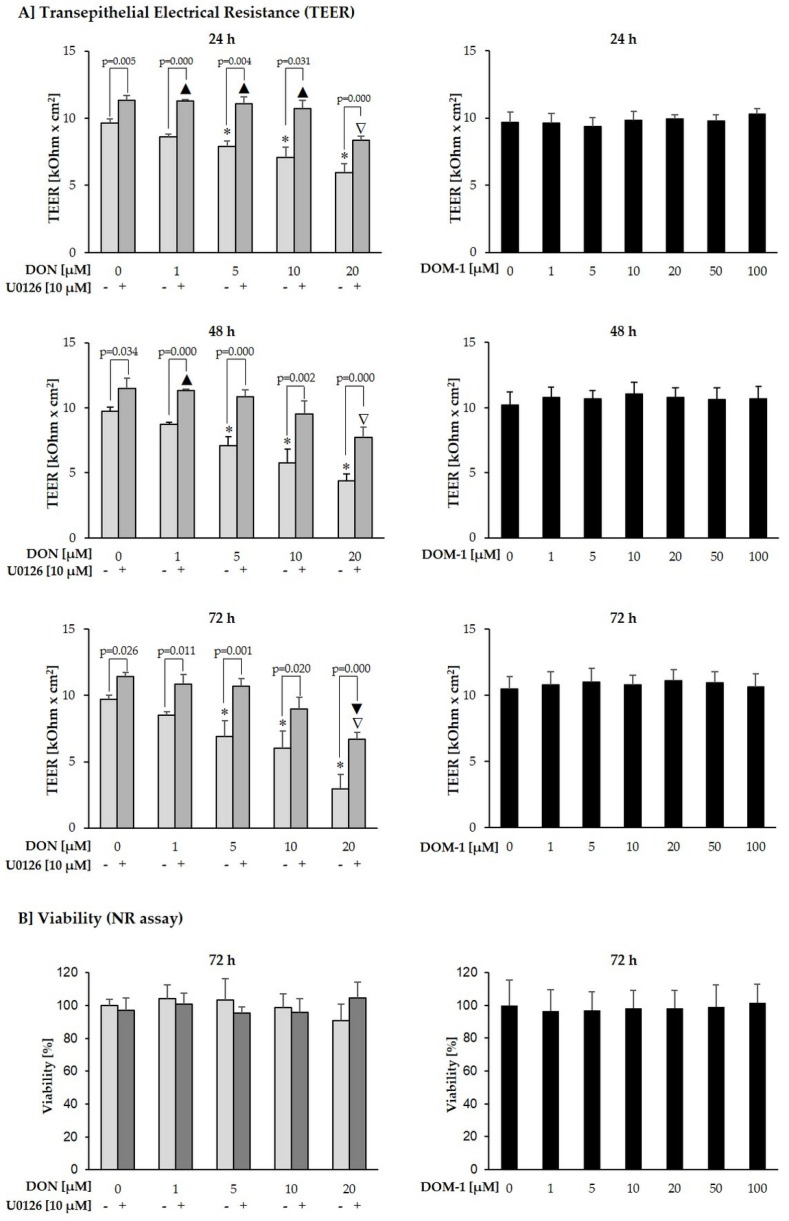
Effect of DON (+/− U0126) and DOM-1 on TEER and viability of differentiated IPEC-J2. Differentiated IPEC-J2 were either pretreated with U0126 (10 µM) or cultivation medium, before addition of DON (1–20 µM). Alternatively, differentiated IPEC-J2 were treated only with DOM-1 (1–100 µM). (**A**) TEER was measured after 24, 48 and 72 h (*p*-values indicate significant differences between IPEC-J2 treated with DON alone or DON + U0126 at individual concentrations; significant differences (*) of DON-treated cells to untreated control; significant increases (▲) or decreases (▼) of DON+U0126-treated cells compared to untreated control; significant decreases (▽) of U0126-treated cells compared to U0126 control. (**B**) Following the final TEER measurement, viability was determined via NR uptake. Data represent mean ± SD, *n* = 4.

Effects of DON (1–20 µM) and DOM-1 (1–100 µM) were investigated on TEER of differentiated IPEC-J2. To explore the involvement of MAPK signaling in DON-induced effects on IPEC-J2 barrier function, cells were additionally pretreated with complete cultivation medium or the p44/42 inhibitor U0126 (10 µM), followed by addition of DON (1–20 µM) (Figure 3). Compared to the untreated control, DON significantly reduced TEER at 5–20 µM after 24 h (5 µM: *p* = 0.042; 10 µM: *p* = 0.006; 20 µM: *p* = 0.000), 48 h (5 µM: *p* = 0.022; 10 µM: *p* = 0.032; 20 µM: *p* = 0.000) and 72 h (5, 10, and 20 µM: *p* = 0.000) (*). After 72 h, TEER reached a minimum of 2.93 ± 1.11 kOhm × cm^2^ at 20 µM DON. However, IPEC-J2 treated with DON+U0126, displayed significant TEER reductions only at 20 µM DON (*p* = 0.002) after 72 h (▲), compared to the untreated control. Compared to the untreated control, TEER of U0126-pretreated cells exposed to DON + U0126, was significantly elevated at 1 µM (*p* = 0.000), 5 µM (*p* = 0.001) and 10 µM (*p* = 0.009) after 24 h and at 1 µM (*p* = 0.007) after 48 h (▼). Compared to the U0126 control at each time point, TEER of cells treated with a combination of DON and U0126 was significantly reduced at 20 µM after 24 h (*p* = 0.000), 48 h (*p* = 0.011) and 72 h (*p* = 0.000) (▽). These differences are due to the fact that the 24 h U0126 pre-treatment alone, already significantly raised TEER values compared to cells pretreated with complete cultivation medium. U0126 pretreatment alone increased TEER of differentiated IPEC-J2 by 1.68 kOhm × cm^2^ after 24 h (+17%; *p* = 0.004), 1.77 kOhm × cm^2^ after 48 h (+18%; *p* = 0.034) and 1.73 kOhm × cm^2^ after 72 h (+18%; *p* = 0.026)), compared to cells which were pretreated with complete cultivation medium. At all individual DON concentrations and time points, TEER of cells treated with a combination of DON and U0126 was significantly higher than that of cells treated with DON alone.

To investigate if and how far TEER reductions were a result of cytotoxic effects, a NR assay was conducted in Transwell^®^ polyester membrane inserts following the final TEER measurement after 72 h. U0126 had no beneficial effect on the viability of DON-treated IPEC-J2. Viability of IPEC-J2 treated with DON (+/− U0126) remained unaffected over the entire concentration range. Thus, DON (1–20 µM) led to significant TEER reductions of differentiated intestinal epithelial cells in a time- and dose-dependent manner, without affecting viability.

In contrast to DON, its de-epoxy metabolite DOM-1 had no negative effect on TEER or viability of differentiated IPEC-J2 up to a concentration of 100 µM, i.e., the five-fold concentration of DON, over a period of 72 h.

### 2.4. Calcium Switch Assay

**Figure 4:**
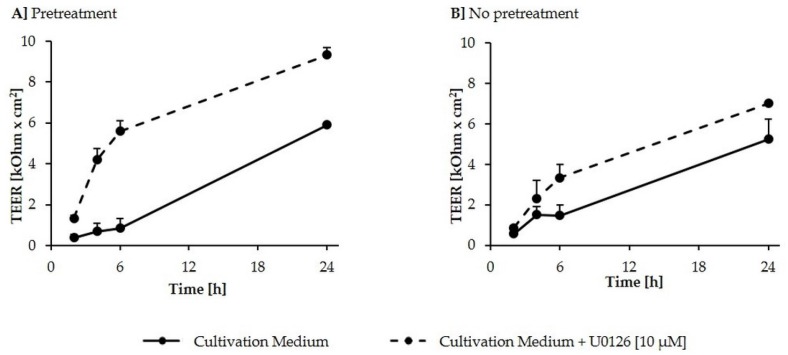
Effect of U0126 (10 µM) on reassembly of a tight IPEC-J2 monolayer after calcium deprivation of differentiated IPEC-J2. IPEC-J2, differentiated in 1.12 cm^2^ non-coated Transwell^®^ polyester membrane inserts, were: (**A**) either pretreated U0126 (10 µM) or cultivation medium before calcium deprivation; or (**B**) exposed to calcium deprivation without pre-treatment. TEER was measured during recovery (0, 2, 4, 6 and 24 h) in complete calcium-containing cultivation medium with or without U0126 (10 µM). Test and control wells were tested in triplicate. Data represent mean ± SD, *n* = 4.

To further investigate the positive effect of U0126 pretreatment on TEER values of untreated IPEC-J2, a calcium switch assay was performed to determine reassembly of a tight IPEC-J2 monolayer after its deliberate destruction (Figure 4). U0126-supplemented cultivation medium, compared to cultivation medium alone, induced a more rapid increase of TEER in the 24 h following calcium deprivation. Even without a 24 h preincubation step with U0126, preceding the calcium switch, U0126 (10 µM) accelerated monolayer formation after calcium deprivation, even if less effectively.

### 2.5. Cytotoxicity

**Figure 5:**
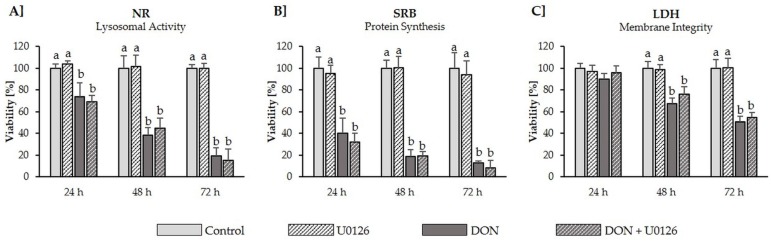
Effect of DON (50 µM) on viability differentiated IPEC-J2 in the absence or presence of MAPK inhibitor U0126 (10 µM) after 24 h, 48 h and 72 h Differentiated IPEC-J2 were either pretreated with U0126 (10 µM) or cultivation medium, before addition of DON (50 µM). Viability was assessed according to the (**A**) neutral red (NR); (**B**) sulforhodamine B (SRB) and (**C**) lactate dehydrogenase (LDH) assay after 24, 48 and 72 h. Data was normalized to control and represent mean ± SD, *n* = 4. Statistically significant differences (*p* < 0.05) are indicated by different letters (a,b).

According to the NR assay performed after the final TEER measurement (Figure 3b), we observed that neither DON (1–20 µM), nor the combination of DON (1–20 µM) + U0126 (10 µM), significantly reduced viability after 72 h. However, to further investigate whether U0126 is able to protect against DON-induced cytotoxicity, a cytotoxic dose of DON (50 µM) was applied to differentiated IPEC-J2 in the absence or presence of U0126. Subsequently, viability was assessed via the NR (lysosomal activity), SRB (total protein content) and the LDH (membrane integrity) assay after 24, 48 and 72 h (Figure 5). According to the NR and SRB assay, DON induced significant viability reductions after 24 h (NR: −27%; SRB: −60%), 48 h (NR: −61%; SRB: −81%) and 72 h (NR: −81%; SRB: −87%). Addition of U0126 did not counteract the cytotoxic effect of DON, producing similar viability reductions after 24 h (NR: −31%; SRB: −56%), 48 h (NR: −56%; SRB: −81%) and 72 h (NR: −85%, SRB: −92%), as cells treated with DON alone. According to the LDH assay, DON (+/− U0126) was not cytotoxic after 24 h. After 48 h and 72 h, viability was significantly reduced in both DON−treated (48 h: −33%; 72 h: −50%) and DON + U0126-treated (48 h: −26%; 72 h: −46%) cells. Thus, U0126 did not counteract or reduce DON−induced toxicity over an incubation period of 72 h, showing a similar time-dependent cytotoxicity profile as DON alone.

### 2.6. Tight Junctions

**Figure 6:**
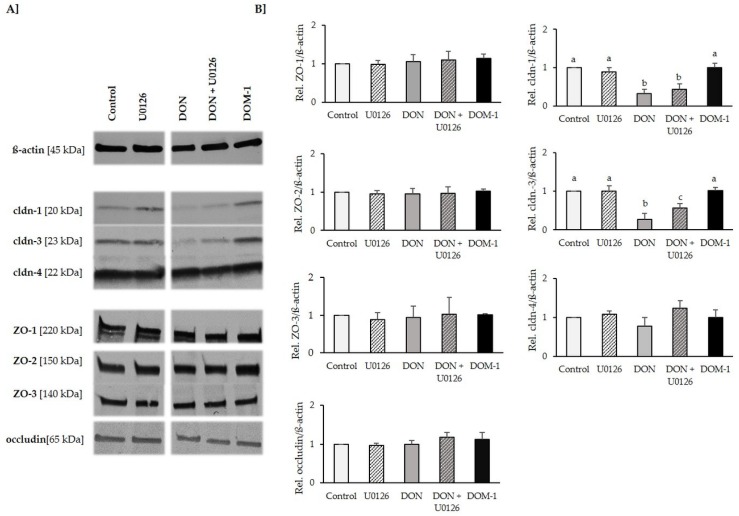
Effect of DON (20 µM) +/− U0126 (10 µM) on tight junction components of differentiated IPEC-J2. Differentiated IPEC-J2 were either pretreated with U0126 (10 µM) or cultivation medium before addition of DON (20 µM) for 72 h. Alternatively, differentiated IPEC-J2 were treated with DOM-1 for 1 h. The expression of tight junction components claudin-1, -3 and -4; ZO-1, -2 and -3; and occludin was determined by (**A**) immunoblotting and (**B**) densitometry after normalization with ß-actin. Data were normalized to control and represent mean ± SD, *n* = 4. Statistically significant differences (*p* < 0.05) are indicated by different letters (a,b,c).

As 20 µM significantly reduced TEER without reducing viability, we examined the effect of this DON concentration on tight junction proteins, in the presence and absence of U0126 (10 µM). Additionally, we tested the effect of DOM-1 (100 µM) on the expression of tight junction proteins claudin-1, -3 and -4; ZO-1, -2 and -3; and occludin (Figure 6). DON did not affect ZO-1, -2 and -3; occludin; or claudin-4, but significantly reduced claudin-1 to 0.32 ± 0.11 (*p* = 0.12) and claudin-3 to 0.27 ± 0.15 (*p* = 0.000), compared to the control. This is equivalent to reductions of 68% and 73%, respectively. Although the significant reduction of claudin-3 expression could be counteracted by U0126, this was not possible for claudin-1. In contrast to DON, DOM-1—even if applied at a five-fold higher concentration—did not affect any of the tested tight junction proteins.

## 3. Discussion

The epithelium lining the gastrointestinal tract separates the external environment from the bodies’ internal milieu and therefore forms an important physical barrier against pathogens and toxic macromolecules. Adjacent epithelial cells are closely linked by an intercellular junctional complex of tight junctions, adherens junctions, gap junctions and desmosomes. Especially tight junctions, which represent a central part of the compartmentalization, are key determinants of paracellular permeability [4,10,26]. Impairments of the intestinal epithelial barrier—as have been reported following exposure to mycotoxins such as DON [8,27]—often facilitate paracellular passage of xenobiotics, harmful molecules and pathogenic microorganisms, which may in turn affect systemic immunity [23].

While the detrimental effect of DON on intestinal barrier integrity as such has been investigated [8,9,10,27,28], details regarding the mechanisms mediating these effects are scarce. Therefore, the present study not only investigated the effects of DON and—for the first time—DOM-1, on barrier integrity and tight junction expression of the differentiated IPEC-J2, it also examined the extent to which detrimental effects of DON are mediated through the activation of MAPK protein p44/42. DON-induced damage of the epithelial layer may partially be due to the fact that intestinal epithelial cells are confronted with high luminal concentrations for lengthy durations. For examining the effect of DON on MAPK activation, TEER and tight junctions, the present study employed DON concentrations ranging up to 20 µM. This range not only correspond to levels of the mycotoxin identified in feed, such as wheat, barley or corn meal, it is also a concentration range that triggers significant intestinal disturbances [9,29,30,31,32].

Pigs, whose diet is largely comprised of cereal-rich feed, exhibit particularly high DON susceptibility [15]. To facilitate analysis of gastrointestinal impairments, a suitable in vitro model is essential, from which results can be extrapolated—at least to a certain extent—to in vivo situations. However, while the use of human and rodent intestinal cell lines—which are often additionally limited due to their tumorigenic origin—has been widely documented, current availability of porcine intestinal epithelial cells is restricted to IPEC-1 [33], IPEC-J2 [34] and IPI-2I [35]. Due to the similarities with the original in vivo porcine cell tissue and with humans, the use of porcine cell lines should be encouraged [25,36]. Both IPEC-1 and IPEC-J2 possess strong morphological and functional resemblance to intestinal epithelial cells in vivo. However, IPEC-J2 were recently reported to be morphologically and functionally even more differentiated than IPEC-1 [37], therefore acting as an even more suitable cellular model. Nevertheless, the majority of studies analyzing the effect of DON on barrier function were performed with the tumorigenic cell line Caco-2 [27,38,39,40,41,42] or the porcine cell line IPEC-1 [8,9,43,44]. Investigations of the effects of mycotoxins on IPEC-J2 however are limited [10,45,46].

To study the impact of DON and DOM-1 on barrier function, IPEC-J2 were differentiated on permeable inserts, thereby promoting the buildup of a polarized epithelial barrier with its differentiated apical and basolateral features [47]. The steady-state TEER phase, as observed between Days 7 and 11, provided suitable conditions for toxin treatment, during which the in vitro situation was assumed to simulate the in vivo epithelial barrier to an acceptable extent. Compared to the tumor cell line Caco-2, which requires up to 30 days for differentiation, IPEC-J2 were originally derived from native intestinal enterocytes [34]. Their fast differentiation could therefore be an inherent enterocytic property, considering that the in vivo lifetime of intestinal enterocytes is restricted to approximately three days [48]. Geens and Niewold [49] reported a tight IPEC-J2 monolayer in 1.12 cm^2^ collagen-coated Transwell^®^ inserts by Day 7, the formation of apical villi, desmosomes, and intercellular spaces by Day 9, and a significant increase of microvilli length and diameter up to Day 11 of cultivation. Furthermore, according to Fromter and Diamond [50], IPEC-J2 with TEER values of approximately 2 kΩ·cm^2^ can be considered tight epithelia.

Several studies have demonstrated involvement of MAPK signaling in the barrier function of tight junctions. For example, through stimuli such as transforming growth factor-beta (TGF-ß) [51,52], epidermal growth factor (EGF) [53], hepatocyte growth factor (HGF) [54], interleukin (IL)-17 [55], endostatin [56], glucocorticoids [57], steroids (e.g., estradiol and dihydrotestosterone) [58,59], and bile [60], the expression of tight junction proteins was promoted, thereby improving barrier function. Others have found that MAPK signaling can perturb barrier function by negatively affecting tight junction proteins through the following stimuli: Ras transfection [61,62], oxidative stress [63,64,65], dissociation factor (DF) [66,67], TGF-ß3, EGF+TGF-ß1 [68], platelet derived growth factor (PLGF)-1 [69], vascular endothelial growth factor (VEGF) [70], mast cell tryptase [71], human immunodeficiency virus transactivator of transcription (HIV-Tat) protein [72,73], HIV protease inhibitor [74], matrix metalloproteinases (MMP) 7 and 9) [75,76] and enteropathogenic Escherichia coli (EPEC) [77]. It is of particular interest that, in all of these events, the involved MAPK was p44/42 (ERK1/2). Therefore, since regulation of tight junction structure and function is often mediated by this MAPK [78], we examined the extent to which the activation of p44/42 mediates barrier disruption of DON. We demonstrated DON-induced activation of MAPK p44/42 (ERK1/2) and MAPK p38 within one hour of exposure. The latter is in accordance with literature, where similar effects in porcine intestinal cells of the ileum and jejunum (IPEC-1) [43], mouse spleen [79] and porcine dendritic cells [40] have been reported. Using U0126, we were able to inhibit DON-induced activation of p44/42, while leaving p38 activation unaffected, thereby demonstrating the high selectivity of U0126 towards p44/42. This is in accordance with Pinton et al. [9], who previously demonstrated the suitability of this inhibitor for studying DON-induced mechanisms. Furthermore, according to Favata et al. [80], MEK affinity of U0126, its selectivity for MEK over other kinases and its cellular efficacy, suggest that this compound presents a powerful tool for in vitro and cellular investigations of mitogen-activated protein kinase-mediated signal transduction.

To investigate whether p44/42 activation mediates barrier effects of DON, differentiated IPEC-J2 were exposed to the mycotoxin in the absence and presence of U0126. In agreement with other studies, apical DON treatment of differentiated IPEC-J2—mimicking the direct contact of the epithelial cells with contaminated food or feed components—time- and dose-dependently reduced TEER, a measure of tight junction integrity of endo- and epithelial monolayers [8,10,81]. According to our study, DON significantly reduced TEER at 5–20 µM after 24, 48 and 72 h. Preincubation of differentiated IPEC-J2 with U0126 and subsequent DON treatment in the presence of the inhibitor significantly reduced the negative effect of DON on IPEC-J2 barrier function. This suggests involvement of MAPK p44/42 (ERK1/2) signaling in DON-induced barrier disruption. However, in contrast to Pinton et al. [9], who concluded that preincubation with 0.5 mM MAPK inhibitor U0126 for 2 h reduces DON-induced alteration of TEER in IPEC-1, our findings suggest that U0126 not only acts directly against the effects of DON, but that pretreatment with the inhibitor strengthens the IPEC-J2 monolayer prior to DON addition. Compared to untreated IPEC-J2, we observed significantly higher TEER values in cells pretreated with U0126 for 24 h. Therefore, we hypothesized that the sole inhibition of p44/42 in differentiated IPEC-J2, already promotes barrier function, thereby increasing TEER and resilience against subsequent DON-induced disruption. Via a calcium switch, we compared the reassembly of the IPEC-J2 monolayer, after its deliberate destruction through Ca^2+^ depletion, in cultivation medium with or without U0126. Thereby we demonstrated that U0126-supplemented cultivation medium, compared to cultivation medium alone, induced a far more rapid increase of TEER in the 24 h following calcium deprivation. This supports the hypothesis that the compound U0126 might not only work directly against DON, but that it potentially strengthens the intestinal barrier, such as to increase its resilience against DON. To ensure that the U0126-induced TEER increase is not an inherent property of the substance U0126, but a result of ERK1/2 inhibition, further inhibitors may be tested. However, since the target of these compounds is the inhibition of ERK1/2 activity, it seems likely that they show a similar TEER increase. This is in accordance with Aggarwal et al. [13], who suggests that ERK activation can lead to a disruption of tight junctions in some epithelial monolayers, whereas it can prevent disruption of tight junctions in other monolayers. Thus, inhibition of ERK most likely directly influences the mechanisms involved in tight junction assembly, thereby explaining the TEER increase of U0126-pretreated cells, compared to cells pretreated with complete cultivation medium only.

Although DON significantly reduced TEER of differentiated IPEC-J2 between 5 and 20 µM, viability was not affected at this concentration range, regardless of whether U0126 was added. This is in accordance with Vandenbroucke et al. [31], who reported a substantially lower DON sensitivity in differentiated compared to undifferentiated IPEC-J2. The authors conclude that, while 0.25–10 µg/mL (~845 nM–34 µM) significantly reduced viability of undifferentiated IPEC-J2 within 24 h according to the NR assay, viability of differentiated IPEC-J2 remained unaffected by this concentration range. Thus, DON significantly reduced TEER, without inducing cytotoxicity, suggesting that measurement of the transepithelial resistance serves as a highly sensitive indicator of DON induced damage. We herewith showed that deterioration of the epithelial network clearly precedes cytotoxic effects and exclude that TEER decreases are merely a result of cell death. Nevertheless, in order to evaluate the potential of U0126-mediated p44/42 inhibition to counteract DON induced cytotoxic insults, we exposed differentiated IPEC-J2 to a cytotoxic concentration of DON (50 µM) in the absence and presence of U0126. Subsequently we evaluated viability after 24, 48 and 72 h, using three viability assays of different endpoints. Interestingly, despite the strong effect observed against DON-induced TEER reductions, U0126 was not able to counteract DON cytotoxicity. This suggests that while barrier disruption is at least partially a downstream effect of p44/42 activation, DON-induced cell death may be mediated through alternative pathways. Possibly, cytotoxicity of DON is a downstream effect of early p38 activation, which is responsible—among other things—for regulating apoptosis. To our knowledge, this is the first study, to evaluate the potential of a MAPK inhibitor to protect against DON cytotoxicity.

The integrity of the epithelial barrier relies on tight junctions. For instance, ZO proteins connect apical membrane proteins, such as occludin and claudins, with proteins of the cytoskeleton, such as actin. Disruption of these actin filaments leads to a collapse of the TJ protein network. This is mirrored in decreases of TEER, an early marker of the integrity of tight junction dynamics. We investigated the effect of DON on tight junction components claudin-1, -3 and -4; ZO-1, -2 and -3; and occludin. Despite the strong effect observed on TEER of DON-treated cells, DON induced significant reductions of claudin-1 and -3 only, while leaving protein levels of claudin-4; ZO-1, -2 and -3; and occludin unaffected. Thus, although a direct effect of DON on TJs is assumed, these findings strongly suggest that the TJ network had not entirely lost its function. This is in accordance with Akbari et al. [27], who demonstrated irregular structures of the stained tight junction proteins in DON-exposed cells, suggesting a clumping and internalization of fragmented networks, rather than a total disappearance. The authors noted that such altered distribution patters may leave the measured total immunoreactive protein level unchanged. Therefore, in addition to measuring total immunoreactive protein, immunofluorescence staining of tight junction proteins would be a suitable method to examine tight junction distribution in response to DON. With respect to our analysis of the involvement of MAPK signaling in the DON-induced reduction of tight junction proteins claudin-1 and -3, we could show that while U0126 was able to at least partially counteract DON-induced claudin-3 reduction, the effects of DON on claudin-1 could not be reversed by inhibition of MAPK p44/42 (ERK1/2).

Regarding the influence of DON on TJ proteins of IPEC-J2, literature reports exist with respect to ZO-1, claudin-3 and occludin [10,45,82,83]. Gu et al. [83] reported ZO-1 reductions after 72 h basolateral exposure to DON (6.73 µM). Others demonstrated—in accordance with our finding—no [45] or only slight [10,82] DON-induced (6.73 µM) ZO-1 reductions in apically treated IPEC-J2, but strong effects in cells exposed basolaterally for 72 h. While our finding of a DON-induced claudin-3 decrease is in accordance with Gu et al. [83], others report no effect of DON on claudin-3 in apically treated IPEC-J2 [10]. Finally, the effect of occludin was studied in DON-treated IPEC-J2, showing—in contrast to our finding—a decrease of the TJ component at 6.73 µM after 48 h [83]. To our best knowledge, we are the first to examine the effect of DON on tight junctions in the morphologically highly representative and well differentiated IPEC-J2. We are therefore the first to report a reduction of claudin-1 in DON-treated IPEC-J2 and the absence of an effect on claudin-4, ZO-2 and -3. With regard to the porcine cell line IPEC-1, Pinton et al. [8] provides valuable knowledge regarding a DON-induced (30 µM) reduction of claudin-3 and -4 within 48 h treatment. Furthermore, the same group reported—in contrast to the present study—the reduction of claudin-4 in IPEC-1 to be mediated through activation of p44/42 [9]. Most likely, this discrepancy is due to the use of different cell lines. While IPEC-1 were originated from the jejunum and ileum, IPEC-J2 were isolated solely from the jejunum. In fact, Nossol et al. [37] performed a comparison of the two cell lines with regard to morphological differentiation, function and metabolism. The authors concluded that of the 14,746 analyzed genes, 5787 differed significantly.

Remaining studies, most frequently employing Caco-2, demonstrated a DON-induced (4.2 µM) reduction of claudin-3 in apically treated cells within 24 h [38], as well as a DON-induced reduction of claudin-1, -3 and -4 and no effect on occludin following a 24 h basolateral exposure [27]. Furthermore, DON (~1.7–17 µM) has been shown to reduce claudin-4 within 24 h, while leaving occludin unaffected [39]. Again others reported DON-induced (30 µM) reductions of claudin-4 within 48 h, while leaving claudin-3 unaffected [8].

Contamination of food and feed is not restricted to DON, but is frequently accompanied by derivatives of the mycotoxin. Although potential toxicological effects of DOM-1 have been poorly documented, its natural exposure and the frequent use of DON to DOM-1 converting feed additives, justifies investigations of the DON-derived deepoxy metabolite. In fact, due to the limited availability of absorption and toxicity data, the amount of DON metabolites has not been considered in the regulatory limits for DON [23]. Hence, the present study included—in addition to the analysis of DON—the first investigation of DOM-1 with respect to porcine intestinal barrier function and tight junction expression. Only few literature references deal with the in vitro effects of DON metabolite DOM-1. Dänicke et al. [84] have evaluated DOM-1 cytotoxicity in porcine PBMCs and undifferentiated IPEC-1 and IPEC-J2. Furthermore, the same group studied the effect of DOM-1 on proliferation of bovine PBMCs [85]. Sundstol-Erriksen et al. [86] tested the effect of DOM-1 on DNA synthesis using 3T3 fibroblasts and reported a 55 time higher IC-50 of DOM-1 compared to DON. Finally, Nasri et al. [87] have assessed the effect of cytotoxic and apoptotic potential of DOM-1 in Jurkat T-cells. In a recently published and highly relevant study, Pierron et al. [88] investigated the molecular basis of DOM-1 reduced toxicity, concluding that bacterial de-epoxidation of DON alters the interaction with the ribosome. According to the authors, both DON and DOM-1 fit into the pockets of the A-site of the ribosomal peptidyl transferase center. However, while DON forms three hydrogen bonds and activates MAPK signaling, DOM-1 forms only two hydrogen bonds and does not activate MAPK signaling. This is in accordance with our finding, that in contrast to DON, even the fivefold concentration of DOM-1, did not phosphorylate either MAPK p44/42 nor p38. Furthermore, DOM-1 had no effect on TEER or viability of differentiated IPEC-J2 over the entire concentration range of 1–100 µM. Finally, DOM-1 did not reduce expression of tight junction proteins claudin-1, -3 and -4; ZO-1, -2 and -3; or occludin. This is in agreement with the above-mentioned studies and supports their findings that the de-epoxidation of DON to DOM-1 deprives the mycotoxin of its pathological effects and partially confirms the safety of feed additives containing DON to DOM-1 transforming bacteria. Furthermore, the experimental model presented in this investigation would be a valuable tool to study the effects of other DON metabolites, such 3- and 15-acetyl-DON, 3-O-glucoside-DON, DON-3 and DON-15-glucuronide, as well as DON-sulfonates. Although these derivatives have been subject to several in vitro studies [43,84,89,90], the current experimental model would be an interesting tool to gain further knowledge regarding their toxicological profile.

## 4. Conclusions

We herewith present a thorough in vitro investigation of the effects of DON and DOM-1 on intestinal barrier integrity. Aside from providing the first in vitro assessment of DOM-1 on the expression of seven TJ proteins, this is the first study to evaluate the effects of DON on claudin-1 and claudin-4 as well as ZO-2 and ZO-3 in the morphologically highly representative porcine cell line, IPEC-J2. We provide information regarding the extent to which the effects of DON are due to the early activation of the MAPK p44/42. We herein report that, in contrast to DOM-1, DON significantly impairs TEER of differentiated IPEC-J2 and selectively affects TJ proteins claudin-1 and -3, while leaving claudin-4; ZO-1, -2 and -3; and occludin unaffected. We demonstrate that while detrimental effects on TEER and claudin-3 are at least partially p44/42 mediated, negative effects on claudin-1 and IPEC-J2 viability are not mediated through this pathway. We are therefore the first to report a connection between DON-induced activation of MAPK p44/42 and claudin-3 reduction. We highlight the significance of this study, considering that an injured epithelial cell barrier may be one of the predisposing factors leading to inflammatory diseases. Considering the high levels of DON contamination in both feed and food, consumption of the latter may have substantial health implications not only for animals, but also humans.

## 5. Materials and Methods

### 5.1. Cell Culture

The intestinal porcine epithelial cells IPEC-J2 (ACC701; Leibniz Institute DSMZ (German Collection of Microorganisms and Cell Cultures, Braunschweig, Germany) were cultured in complete cultivation medium consisting of Dulbecco’s modified eagle medium (DMEM)/Ham’s 12 (1:1) (Biochrom AG, Berlin, Germany), supplemented with 5% fetal bovine serum, 1% insulin-transferrin-selenium, 5 ng/mL epidermal growth factor, 2.5 mM Glutamax (all Gibco™, Life Technologies, Carlsbad, CA, USA) and 16 mM 4-(2-hydroxyethyl)-1-piperazineethanesulfonic acid (Sigma-Aldrich, St. Louis, MO, USA) at 39 °C and 5% CO_2_. Mycoplama tests were performed regularly via PCR to confirm that cells were free of mycoplasma contamination (Venor^®^ GeM Mycoplasma Detection Kit; Minerva Biolabs, Berlin, Germany). For routine cultivation, cells were seeded at 5 × 10^4^ cells/mL in 150 cm^2^ tissue culture flasks (Eppendorf, Hamburg, Germany) with 28 mL complete cultivation medium and subcultured upon confluence every four days for a maximum of 15 passages.

### 5.2. Compounds

Deoxynivalenol (DON) (Biopure, Romer Labs^®^, Tulln, Austria) was dissolved in sterile distilled water to produce a stock solution of 6.75 mM and was further diluted to the desired working concentrations in complete cultivation medium. De-epoxy-deoxynivalenol (DOM-1) (Biopure, Romer Labs^®^, Tulln, Austria) was obtained as liquid calibrant solution (180.15 µM in acetonitrile). Required volumes were evaporated to dryness under nitrogen gas. Depending on the desired working concentration, the remaining DOM-1 was taken up in appropriate volumes of complete cultivation medium. MAPK inhibitor U0126 monoethanolate (Sigma-Aldrich, St. Louis, MO, USA), referred to as U0126, was dissolved in dimethyl sulfoxide (DMSO) to produce a stock solution of 10 mM and was further diluted in complete cultivation medium.

### 5.3. Measurement of Transepithelial Electrical Resistance (TEER)

IPEC-J2 were seeded in the apical compartment of 1.12 cm^2^ Transwell^®^ polyester membrane inserts with 0.4 µm pores (Corning Inc., New York, NY, USA) at a seeding density of 1 × 10^5^ cells/insert. To determine resistance maximum and steady state resistance, TEER measurements were conducted over a period of 30 days after seeding, using a Millicell-Electrical Resistance System (ERS) (Merck Millipore, Billerica, MA, USA). TEER values were calculated as kOhm × cm^2^.

For subsequent experiments, cells were differentiated for seven days and subsequently pretreated with either complete cultivation medium or U0126 (10 µM) for 24 h, followed by the addition of DON (1–20 µM) (Day 8), in the presence (pretreated cells) or absence (not pretreated cells) of MAPK inhibitor U0126 (10 µM) for a total of 72 h. Cells pretreated with cultivation medium (untreated cell control) or cells treated with U0126 (10 µM) (U0126 control) served as negative controls throughout the assay. In an alternative test set-up, IPEC-J2 were differentiated for eight days and subsequently exposed to DOM-1 (both: 1–100 µM) for a total of 72 h. In both cases, TEER was measured at 24 h intervals.

Following the final TEER measurement, viability of IPEC-J2 was examined via the neutral red (NR) assay (Aniara, West Chester, OH, USA). The NR assay assesses the ability of cells to incorporate and bind the supravital dye NR within anionic sites of the lysosomal matrix. At the end of the 72 h incubation period, treatments were removed and cells washed with the washing buffer provided by the assay kit. According to the manufacturer’s specifications, NR was added to the negative control and test inserts at a final concentration of 1:100 in complete cultivation medium for 3 h. Subsequently cell were fixed for 1 min. Incorporated dye was solubilized in and supernatants transferred to a fresh 96-well plate. The quantity of dye incorporated into cells was measured photometrically at 540 nm, with a reference filter of 690 nm (Biotek Instruments Inc., Winooski, VT, USA). The obtained optical density (OD) is directly proportional to the number of viable cells.

### 5.4. Calcium Switch Assay

IPEC-J2 were seeded on 1.12 cm^2^ Transwell^®^ polyester membrane inserts (Corning Inc., New York, NY, USA) at 1 × 10^5^ cells/insert and differentiated for eight days. Cells were then either pretreated or not pretreated with U0126 (10 µM) for 24 h. Subsequently, IPEC-J2 were deprived of calcium by washing twice with Ca^2+^- and Mg^2+^-free Hank’s Balanced Salt Solution (HBSS) (Gibco, Life Technologies, Carlsbad, CA, USA) and exposed to 2 mM ethylene glycol-bis(2-aminoethyl ether)*N*,*N*,*N*′,*N*′-tetraacetic acid (EGTA) (Sigma-Aldrich, St. Louis, MO, USA) in Ca^2+^- and Mg^2+^-free HBSS (Gibco, Life Technologies, Carlsbad, CA, USA) for 10 minutes. HBSS-EGTA was then removed and cells were rinsed with phosphate buffered saline (PBS) and allowed to recover in either complete cultivation medium or in cultivation medium supplemented with U0126 (10 µM). TEER was monitored after 2, 4, 6 and 24 h during this recovery period as an index of tight junction reassembly and restoration of barrier function.

### 5.5. Cytotoxicity Assays

IPEC-J2 were seeded in 96-well plates (Eppendorf, Hamburg, Germany) at 3 × 10^4^ cells/well and differentiated for eight days, with regular medium changes every 48 h. Cells were then either pretreated with complete cultivation medium or U0126 (10 µM) for 24 h, followed by the addition of DON (50 µM) in the presence or absence of MAPK inhibitor U0126 (10 µM) for a total of 72 h. IPEC-J2 viability was then assessed after 24, 48 and 72 h on the basis of lysosomal function (neutral red (NR) assay), total protein content (sulforhodamine B (SRB) assay) and cell membrane integrity (lactate dehydrogenase (LDH) assay).

For the NR assay (Aniara, West Chester, OH, USA), treatments were removed and cells were washed with the washing buffer provided by the assay kit. Subsequently, NR solution (1:100 in cell culture medium) was added to the cells for 3 h at 39 °C and 5% carbon dioxide. Supernatants were then removed and cells fixed for 1 min. Incorporated dye was solubilized and measured photometrically at 540 nm, with a reference filter of 690 nm.

For the SRB assay (Aniara, West Chester, OH, USA), cells were fixed and incubated with SRB for 15 min, excess dye was removed by washing and incorporated dye was dissolved and measured photometrically at 490 nm, with a reference filter of 690 nm.

For the LDH assay (Thermo Scientific Inc., Waltham, MA, USA), 50 µL of cell supernatant was incubated with 50 µL of LDH reaction mixture in a fresh 96-well plate for 30 min. Subsequently, the reaction was stopped and ODwas measured at 490 nm, with a reference filter of 690 nm.

### 5.6. Cell Protein Extraction, SDS-PAGE and Western Blotting

Immunoblot assays were conducted to examine the phosphorylation status of MAPK proteins p44/42 and p38 as well as the expression of tight junction proteins claudin-1, -3 and -4; zona occludens (ZO)-1, -2 and -3; and occludin. In both cases, IPEC-J2 were seeded in 24 mm Transwell^®^ polyester membrane inserts (Corning Inc., New York, NY, USA), differentiated and subsequently pretreated or not pretreated with U0126 (10 µM) for 24 h. Cells which were pretreated with U0126 were then exposed to DON (20 µM) in the presence of U0126, while cells which were not pretreated with U0126 were treated with DON (20 µM) in the absence of U0126. In parallel, eight-day differentiated IPEC-J2 were treated with DOM-1 (100 µM). After 1 h (p44/p42 and p38) or 72 h (tight junction proteins), supernatants were removed and cells were washed twice with ice-cold PBS (Sigma-Aldrich, St. Louis, MO, USA) and incubated with 250 µL radioimmunoprecipitation assay buffer (RIPA) buffer (Sigma-Aldrich, St. Louis, MO, USA) supplemented with 1× complete™ mini Protease Inhibitor Cocktail (Roche, Rotkreuz, Switzerland) for 5 min. Cells were harvested by scraping, transferred to microcentrifuge tubes (Eppendorf, Hamburg, Germany) and incubated for 30 min on ice. Cells were centrifuged (10,000× *g*, 30 min, 4 °C) and supernatants collected. Protein concentration was determined via the BCA assay (ThermoScientific, Waltham, MA, USA).

Samples (10 µg) were loaded on sodium dodecyl sulfate (SDS) polyacrylamide gels (Bio-Rad Laboratories Inc., Hercules, CA, USA) in parallel with the pre-stained ladder (SM1811, 10–250 kD; Fermentas, Darmstadt, Germany) and subsequently transferred onto a polyvinylidene fluoride (PVDF) membrane by semi-dry electroblotting. Membranes were blocked in 5% *w*/*v* skimmed milk power (Sigma-Aldrich, St. Louis, MO, USA), 1× Tris-buffered saline (TBS), 0.1% Tween^®^20 (Sigma-Aldrich, St. Louis, MO, USA) for 1.5 h at room temperature. For MAPK analysis, membranes were probed with rabbit anti-phospho-p44/42 ERK MAPK (1:1000), rabbit anti-endogenous-p44/42 ERK MAPK (1:1000), rabbit anti-phospho-p38 MAPK (Thr180/Tyr182) (3D7), rabbit anti-endogenous-p38 (1:1000) (all Cell Signaling, Danvers, MA, USA) in 5% *w*/*v* bovine serum albumin (BSA), 1× TBS, 0.1% Tween^®^20 overnight (4 °C while gently shaking). For tight junction analysis, membranes were probed with rabbit anti-claudin-1 (D5H1D) XP^®^ (1:2000, Cell Signaling, Danvers, MA, USA), rabbit anti-claudin-3 (1:2000), mouse anti-claudin-4 (1:2000) (ThermoScientific, Waltham, MA, USA), rabbit anti-ZO-1 (D7D12) (1:2000), rabbit anti-ZO-2 (1:2000), rabbit anti-ZO-3 (D57G7) XP™ (1:2000) (Cell Signaling, Danvers, MA, USA), and rabbit anti-occludin (6H10L9) (ThermoScientific, Waltham, MA, USA) in 5% *w*/*v* BSA, 1× TBS, 0.1% Tween^®^20 (all Sigma-Aldrich, St. Louis, MO, USA), overnight at 4 °C while gently shaking. In all experiments, detection of ß-actin with (13E5) rabbit monoclonal antibody (1:2000; Cell Signaling, Danvers, MA, USA) was used as internal loading control. Following incubation, membranes were washed and incubated with alkaline phosphatase labeled goat anti-rabbit IgG (Sigma-Aldrich, St. Louis, MO, USA) or in case of claudin-4 detection with alkaline phosphatase labelled goat anti-mouse IgG (Sigma-Aldrich, St. Louis, MO, USA) for 1.5 h at room temperature while gently shaking. After washing, blots were developed in substrate buffer (100 mM Tris pH 9.5, 100 mM NaCl, 5 mM MgCl_2_ (all Sigma-Aldrich, St. Louis, MO, USA)) supplemented with 5-Bromo-4-chloro-3-indolyl phosphate disodium salt (BCIP) and Nitro-blue tetrazolium chloride (NBT) (both ThermoScientific, Waltham, MA, USA). Membranes were analyzed using myImageAnalysis™ Software (Version 2.0, Thermo Fisher Scientific, Waltham, MA, USA, 2014).

### 5.7. Statistical Analysis

Statistical analysis was performed with IBM^®^ SPSS Statistics (Version 19.0, IBM corp., New York, NY, USA, 2010). Values of each independent experiment were expressed as means of triplicates ± standard deviation (SD). All values were analyzed for normality (Shapiro–Wilk) as well as homogeneity of variance (Levene Statistics). Normally distributed homogenous data were analyzed by analysis of variance (ANOVA) and the Dunnett’s t-test compared to the control or by the Bonferroni test to compare two groups. If data were normally distributed but not homogenous, ANOVA and Dunnett’s T3-test was used. If normal distribution was violated, the Kruskall–Wallis Test was used.

## Abbreviations

The following abbreviations were used in this manuscript:

BCA	bicinchoninic acid
BCIP	5-Bromo-4-chloro-3-indolyl phosphate
BSA	bovine serum albumin
Cldn	claudin
DF	dissociation factor
DMEM	Dulbecco’s modified Eagle’s medium
DMSO	dimethyl sulfoxide
DOM-1	deepoxy deoxynivalenol
DON	deoxynivalenol
EGF	epidermal growth factor
EGTA	ethylene glycol-bis(β-aminoethyl ether)-*N*,*N*,*N*′,*N*′-tetraacetic acid
EPEC	enteropathogenic Escherichia coli
ERS	Electrical Resistance System
FBS	fetal bovine serum
HBSS	Hank’s balanced salt solution
HGF	hepatocyte growth factor
HIV	human immunodeficiency
IL	interleukin
IPEC	Intestinal porcine epithelial cells
ITS	insulin-transferrin-selenium
EGF	epidermal growth factor
LDH	lactate dehydrogenase
MAPK	mitogen activated protein kinase
MMP	matrix metalloproteinases
NBT	Nitro blue tetrazolium
NR	neutral red
OD	optical density
PBS	phosphate buffered saline
PDGF-1	platelet derived growth factor-1
RIPA	Radioimmunoprecipitation assay
PVDF	Polyvinylidene fluoride
SDS	Sodium dodecyl sulfate
SRB	sulforhodamine B
TBS	Tris-buffered saline
TEER	transepithelial electrical resistance
TGF-ß	transforming growth factor-beta
VEGF	vascular endothelial growth factor
ZO	zona occludens
